# Feedback versus no feedback in improving patient outcome in group psychotherapy for eating disorders (F-EAT): protocol for a randomized clinical trial

**DOI:** 10.1186/1745-6215-15-138

**Published:** 2014-04-23

**Authors:** Annika Helgadóttir Davidsen, Stig Poulsen, Mette Waaddegaard, Jane Lindschou, Marianne Lau

**Affiliations:** 1 Stolpegaard Psychotherapy Centre, Stolpegaardsvej 20, Gentofte 2820, Denmark; 2 Department of Psychology, University of Copenhagen, Øster Farimagsgade 2A, Copenhagen K 1353, Denmark; 3 Copenhagen Trial Unit, Centre for Clinical Intervention Research, Rigshospitalet, Copenhagen University Hospital, Blegdamsvej 9, Copenhagen, OE 2100, Denmark

**Keywords:** Psychotherapy research, Outcome, Feedback, Eating disorders, Group psychotherapy, Attendance, Dropout

## Abstract

**Background:**

Continuous feedback on patient improvement and the therapeutic alliance may reduce the number of dropouts and increase patient outcome. There are, however, only three published randomized trials on the effect of feedback on the treatment of eating disorders, showing inconclusive results, and there are no randomized trials on the effect of feedback in group therapy. Accordingly the current randomized clinical trial, initiated in September 2012 at the outpatient clinic for eating disorders at Stolpegaard Psychotherapy Centre, aims to investigate the impact of continuous feedback on attendance and outcome in group psychotherapy.

**Methods/design:**

The hypothesis is that continuous feedback to both patient and therapist on treatment progress and alliance will increase attendance and treatment outcome. The trial is set up using a randomized design with a minimum of 128 patients allocated to either an experimental or control group at a ratio of 1:1. The experimental group will receive standard treatment (systemic and narrative group psychotherapy) with feedback intervention, whereas the control group will receive standard treatment only. The participants are diagnosed with bulimia nervosa, binge eating disorder, or an eating disorder not otherwise specified, according to the DSM-IV. In the experimental group feedback to the participants, based on the Outcome Rating Scale (ORS) and the Group Session Rating Scale (GSRS), is actively added to standard treatment. The ORS assesses areas of life functioning known to change as a result of therapeutic intervention. The GSRS assesses key dimensions of effective therapeutic relationships. In the control group, the patients fill out the Outcome Rating Scale only, and feedback is not provided.

The primary outcome is the rate of attendance to treatment sessions. The secondary outcome is the severity of eating disorder symptoms. Exploratory outcomes are the level of psychological and social functioning, and suicide or self-harm. This is measured with the ORS, Symptom Check List, WHO-Five Wellbeing Index, Sheehan Disability Scale and a modified version of the Self-Harm Inventory.

**Discussion:**

If the results will confirm the hypothesis, this trial will support feedback as a way to improve group treatment attendance for outpatients with eating disorders.

**Trial registration:**

ClinicalTrials.gov identifier: NCT01693237

## Background

Eating disorders are serious mental disorders affecting up to 10% of the population, primarily women [1,2]. Characteristics of eating disorders are severe disturbances in eating behaviour [3] with significant physical, psychological and social consequences for the individual and his or her relatives. According to the DSM-IV, there are two specific diagnoses: anorexia nervosa (AN) and bulimia nervosa (BN). Disorders that do not meet the criteria for a specific eating disorder are classified as eating disorders not otherwise specified (EDNOS) [3]. However a third diagnosis, binge eating disorder (BED), is included in the appendix of the DSM-IV as a ‘diagnosis for further study’. We are diagnosing BED according to the research criteria defined in DSM-IV. In the fifth version of the DSM, BED is recognized as a free-standing diagnosis [4].

### Dropout and non-attendance in psychotherapy

The term ‘dropout’ refers to patients that choose to end treatment prematurely. It is a common phenomenon in psychotherapy. A recent comprehensive meta-analysis, analyzing 669 studies and 83,834 adult patients with a non-psychotic disorder, reported that, on average, 19.7% of patients dropped out of psychotherapy. The dropout proportion for a subsample of patients with eating disorders was even higher at 23.9% [5].

Previous research has indicated that patients who discontinue treatment prematurely exhibit poorer treatment outcomes [5,6]. In group therapy especially, dropout not only affects the patients themselves and their therapists, but can also have an adverse effect on the remaining group members, sometimes resulting in a ‘wave effect’ with dropouts begetting other dropouts [6].

Related to dropout is the phenomenon of irregular attendance to treatment sessions. It has been demonstrated that psychotherapy attendance is associated with the outcome of treatment [7-9] and, accordingly, the development and testing of interventions to increase attendance and prevent dropout from psychotherapy is highly relevant.

### Feedback-informed psychotherapy

One of the ways to specifically address non-attendance and dropout in psychotherapy is by monitoring patient progress. Since 1996, when Howard *et al.*[10] published their session-to-session measures of client progress, there has been an increasing interest in this method and there are now several outcome monitoring systems available [11-13].

Most of the effectiveness research of feedback-informed psychotherapy is based on the Outcome Questionnaire System (OQ System) [11,12,14,15] and the Partners for Change Outcome Management System: International Center for Clinical Excellence (PCOMS ICCE) [12,16-18]. Both systems are based on a patient-focused perspective, where the patient’s goals, ideas for change, and perceptions of the therapeutic alliance are a focus rather than the specific treatment [16,19].

In the remainder of this paper, we will refer to PCOMS ICCE as ‘feedback-informed treatment’ (FIT)*.* FIT is defined as ‘a pantheoretical approach for evaluating and improving the quality and effectiveness of behavioral health services’ [18]. By filling out the measures, the patient continuously evaluates the therapeutic alliance and outcome. This feedback allows the therapist to monitor the progress from session to session and to tailor the treatment in dialogue with the patient. FIT consists of the Outcome Rating Scale (ORS), the Session Rating Scale (SRS) and the Group Session Rating Scale (GSRS) [17]. Information about FIT and intervention strategies are accessible in six different manuals [20]. In January 2013, FIT was recognized as an evidence-based practice by the National Registry of Evidence-based Programs and Practices [21].

### Previous research on the use of feedback in psychotherapy of eating disorders

Results from previous meta-analyses have indicated that feedback has a significant positive effect on psychotherapy outcome [11,22,23]. However, none of these reviews have reported results for subgroups of patients with eating disorders. We therefore searched PsycInfo, PubMed, Embase and the Cochrane Library on 1 October 2013 with the search terms eating disorder*, bulimi* nervosa, anorexi* nervosa, binge eating disorder*, EDNOS and feedback, routine outcome monitoring, and tracking. From the search, we identified three randomized trials [24-26] which we will review in the following.

Schmidt *et al.*[24] included 61 women with BN or EDNOS. They were randomly allocated to 14 sessions of cognitive behavioral guided self-care with or without added personalized feedback. The authors found no effect of feedback on treatment dropout (*P* = 0.672) but it had a significant effect on dietary restriction (*P =* 0.03).

Truitt *et al.*[26] included 51 women with AN or BN and randomly assigned them to treatment with or without feedback. In this trial, the feedback (therapists received feedback, patients did not) significantly predicted change in individual global psychological dysfunction (measured with the OQ System) across the course of treatment (*P* = 0.017).

Simon *et al.*[25] included 133 women diagnosed with AN, BN or EDNOS in their trial. Patients were randomly assigned to treatment with or without feedback using a randomized block design, with therapists serving as the blocking variable (the same therapists provided both treatments). The patients used the OQ System, which, together with Body Mass Index (BMI), was used as an outcome measure. The difference between the feedback condition and no feedback condition at end of treatment was statistically significant and showed an effect size difference of Cohen’s *d* = 0.30. The feedback did not have a significant effect on BMI, which was against the researchers’ initial expectations.

The results in the Schmidt *et al.* and Truitt *et al.* trials [24,26] were challenged by small sample sizes (N = 61 and N = 51), which limits the generalizability and increases the risk of random errors. In the Truitt *et al.* trial [26], the treatment intervention was not described in the dissertation, which makes the trial impossible to repeat. The feedback method in the Schmidt *et al.* trial [24] was defined as ‘personalized feedback on current physical and psychological status, risk and problems, and variables facilitating or hindering change’. The feedback differs from the OQ System and FIT in frequency (not used regularly during therapy) and seems to focus primarily on symptom status rather than on the therapeutic process. These differences reduce the external validity of the findings. The different outcome measures used in the trials can yield different results: Schmidt *et al.* only reported eating disorders symptoms; Truitt *et al.* reported psychological dysfunction in more broad terms (including the OQ score); and Simon *et al.* used the OQ score and BMI as outcome measures.

Previous research has indicated that feedback has a promising effect on maximizing the benefits of psychotherapy. Because only three of the identified trials (with different designs, feedback measures and/or timing and outcome measures) included patients with eating disorders [18,19,21], the benefits of feedback are still inconclusive for these patients. Furthermore, none of the trials were performed in a group therapy setting. 

### Objective

The trial’s objective is to examine the benefits and harms of continuous feedback on treatment progress and alliance in group therapy to patients and therapists. We hypothesize that continuous feedback with subsequent adjustments of the treatment, will increase treatment attendance and treatment outcome.

## Methods/design

The current trial is an investigator-initiated, randomized clinical superiority trial. The experimental intervention is the addition of feedback to standard treatment, and the design strategy can thus be categorized as additive or constructive [27].

After the patients have been offered treatment, received information about the trial, and signed the consent form, the patients are allocated to one of two groups (at a ratio of 1:1). The first (experimental) group consists of standard treatment with feedback intervention (feedback: ORS and GSRS are filled out and included in the treatment according to the FIT intervention manual). The second (control) group consists of standard treatment without feedback intervention (no feedback: ORS is filled out but not included in the treatment).

### Participants

#### ***Selection and withdrawal of participants***

The inclusion and exclusion criteria are divided into criteria that are part of the clinical routine at the trial site, and criteria that are exclusive to the trial (Table 1). Patients excluded as part of the clinical routine will be referred to treatment elsewhere or back to the referring professional. Patients that are offered treatment but are not eligible for inclusion in the trial will receive standard treatment. Excluded patients can be reassessed and included in the trial at a later point in time, if, for example, they have been successfully treated for severe depression and subsequently fulfil the inclusion criteria.

**Table 1:** Inclusion and exclusion criteria

	**Clinical routine**	**Trial specific**
**Inclusion criteria**	• Aged 18 or older	• BN, BED or EDNOS is the primary diagnosis
	• Body Mass Index (BMI = kg/m^2^) ≥ 20	• Diagnosis according to the DSM-IV
		• Written informed consent
**Exclusion criteria**	• Acute suicidal risk	• Severe or non-regulated physical co-morbidity
	• Psychosis	
	• Severe depression	• Pregnancy
	• Abuse of alcohol, medicine and/or narcotics up to 3 months before referral	• Unable to understand Danish
		• Previous participation in the current trial
	• Use of cannabis once a month is accepted at intake but must stop during treatment	• Considered unable to attend treatment sessions as planned
		• Lack of informed consent
	• Concomitant psychotherapeutic/psychiatric treatment outside Stolpegaard Psychotherapy Centre	

BED, binge eating disorder; BMI, Body Mass Index; BN, bulimia nervosa; EDNOS, eating disorder not otherwise specified.

### Participant withdrawal

Participation in the trial is voluntary and patients can withdraw from the trial at any time, without any implications for current or future treatment at Stolpegaard Psychotherapy Centre. The participants are kindly asked to specify which aspects of the trial they wish to withdraw from: the intervention, participation in follow-up assessments, or complete withdrawal from the trial. The options are described in the written patient information.

### Informed consent

Information about participation in the trial will be given at three different points before the patients start therapy: 1) written information before the first routine assessment interview; 2) verbal information at a routine assessment with a psychiatrist or an attending physician; and 3) verbal information at an assessment session with the principal investigator or a qualified assistant. The participants can give their written consent at any point before randomization.

### Therapists

Fifteen therapists are participating in the trial, three male and twelve female (mean age = 44.3 years, SD 9.1). Their general average experience with psychotherapy is 7.2 years (SD 6.6), and their average experience with the treatment of eating disorders is 3.8 years (SD 5.2).

As part of the clinical routine, the therapists have case supervision in groups with team colleagues every other week, lasting one and a half hours. The supervision format is systemic and narrative, structured around the idea of ‘reflecting teams’ [28].

Before data collection began, the therapists had two training sessions (three hours each) in feedback-informed clinical work, led by an external certified FIT trainer and associate at the International Center for Clinical Excellence [20]. The therapists were introduced to the method and instructed in the use of the measures in clinical practice [17]. Only two had worked with FIT in previous jobs. Each therapist was placed in both a control group and an experimental group and thus provided both treatments. To ensure that the therapists followed the feedback method and to reinforce the implementation process, the training was supplemented with one and a half hour hour monthly FIT supervision during the data collection period.

The therapists were assumed to be following the feedback method adequately after this, with no further control for adherence to the method. Because of the lack of published guidelines for the use of feedback in group therapy, the therapists were encouraged to use the feedback according to the guidelines for individual therapy [17]. In cases where these guidelines were insufficient, the supervisor was involved.

Immediately after the second training session with the FIT trainer, the therapists answered an attitude survey (Davidsen, AH; allegiance measure; unpublished data) asking if they believed working with GSRS and ORS would make a positive difference in their therapeutic work. All therapists agreed that it would improve their clinical work. This therapist allegiance can potentially positively influence the results and exaggerate an effect of feedback on outcome. In order to investigate if the first attitude survey was influenced by the ‘rush’ after the training session, we asked the therapists again before they started using the method. The answers were the same in the second survey (Davidsen, AH; allegiance measure; unpublished data).

### Standard treatment

Standard treatment is offered in both groups, based on recommendations from the Danish National Board of Health [2] and on guidelines for treating eating disorders in the Capital Region of Denmark [29]. After the initial assessment interview a psychiatrist or attending physician decides to offer the patient one of two standard treatments: basic or elaborate. This decision is based upon an assessment of eating disorder severity, co-morbidity, and medical and/or social factors that are believed to complicate the treatment of the eating disorder.

Treatment length (from the first assessment session to the last follow-up session) is approximately 10 months for basic treatment and 12 to 14 months for elaborate treatment [29]. After referral and before starting group therapy, the patient undergoes a psychiatric, medical, and somatic assessment, followed by psycho-education. The treatment program consists of 20 sessions for patients with BN or EDNOS, and 25 sessions for patients with BED. Alongside group therapy, the patients are offered sessions with a dietician and relatives as well as sessions with a social worker (as needed). The patients in the elaborate treatment are offered an extended medical assessment and more sessions with relatives and a dietician. In some cases, patients start psychotropic drugs before or during treatment; this is a possibility in both groups. Patient treatment status is assessed at weekly team conferences.

### Therapeutic approach and elements

The eating disorders treatment provided at Stolpegaard Psychotherapy Centre is founded on systemic and narrative theory and a post-modern view on patients and therapy [30-33]. The therapists work with individual therapy in the group, which is structured around the principles of the reflecting team [28]. One of the methods used in the therapy is externalization of the eating disorder [30]. With a focus on the patients’ resources, relations, future and unique outcomes [34], patients and therapists address psychological, physiological and social difficulties associated with the eating disorder. The effectiveness of this specific treatment has not been evaluated but a recent meta-analysis of 38 randomized controlled trials showed that systemic therapy, on which the treatment at the Stolpegaard Psychotherapy Centre outpatient clinic is based, is an effective psychotherapeutic treatment for adults with eating disorders and other psychiatric disorders [35].

### Structure

There are seven patients and two therapists in each group. The groups are slow-open, that is, open to new patients as others end treatment. Central to the treatment is a food diary that patients are asked to keep and discuss in the group therapy sessions. Patients are weighed before each session and weight fluctuation is monitored and addressed in the case of rapid weight gain or weight loss. The patients are encouraged to set individual goals for the treatment, typically concerning food, body and appearance, relations, and future [36]. The duration of a group session is 150 minutes.

### Experimental group (feedback)

In the experimental group two sets of measures are added to the standard treatment: ORS and GSRS.

The patients mark their scores on the ORS and GSRS using a tablet computer with a computer-based application (FIT-Outcomes). FIT-Outcomes (FIT-Outcomes, Hoersholm, Denmark) is an application used to administer, score and aggregate data from the ORS and SRS/GSRS [37]. When the patient has marked his or her scores on the tablet computer, the management system produces a graph illustrating the therapeutic progress on the ORS and GSRS, the cutoff scores and the expected treatment response (ETR). Based on the latest score on the ORS and GSRS, FIT-Outcomes provides immediate feedback to the therapists. The therapists discuss the ORS and GSRS score with the patient in the present or the following group session [17].

### The outcome rating scale (ORS)

Before they are weighed and immediately before each group session, the patients complete the ORS and assess their individual, interpersonal, social, and overall wellbeing during the preceding week. The clinical cutoff score is 25 and the reliable change index (RCI) is 5 points [17]. A green alert in the FIT-Outcomes system means that the patient is on track, that is, that the latest score indicates progress similar to successful treatment courses. A red alert means that the patient is off track, that is, that the latest score indicates progress similar to unsuccessful treatment courses. A yellow alert means an uncertain tendency (that the change is smaller than expected). The therapists are encouraged to discuss yellow and red feedback with the patients as soon as possible [17].

### The group session rating scale

The patients mark a score on the GSRS 5 to 10 minutes before the group session ends, and the therapists respond briefly to the score. On the GSRS, the patients rate the present group session concerning relationship*,* goals and topics*,* approach or method and overall. The clinical cutoff score is 36. As described for the ORS, a green alert from FIT-Outcomes means that there is no reason for an intervention. A red alert encourages the therapists to consider alliance feedback, either due to a below cutoff score or a drop on the GSRS of more than one point. The therapists are encouraged to discuss red feedback in the present or following group session.

### Standard treatment (no feedback)

In the control group*,* the patients fill out a paper version of the ORS before each group session. However, they will not receive any feedback during therapy. The ORS forms are gathered in sealed envelopes and put away until data analyses.

### Outcomes

The primary outcome is treatment attendance in the intervention period, defined as a rate (number of attended therapy sessions divided by the number of planned therapy sessions). If a patient ends therapy prematurely, and this is in accordance with the therapists, the number of attended sessions will be set equal to the planned number of sessions. The secondary outcome is the global score of eating disorder examination (EDE) interview, assessed at the end of intervention. The mean score in the two intervention groups are compared.

The exploratory outcomes are: ORS, the Symptom Check List (SCL-90R), the Sheehan Disability Scale (SDS), the WHO-Five Wellbeing Index (WHO-5), and suicide and self-harm.

For all outcomes except the ORS, the scores obtained in the two intervention groups after the end of the treatment are compared. With regard to the ORS, the last available score for each patient in the two groups is compared (see further description of these outcomes below).

### Assessments

Demographic variables (age, sex, social conditions, education, connection to the labor market), previous treatment, medication, use of alcohol and/or narcotics, BMI, pregnancy, ability to attend treatment, and language skills are assessed at the first assessment interview with a therapist and by a psychiatrist or attending physician at their assessment interview (part of the clinical routine).

Eating disorder diagnosis is assessed at the trial assessment interview. Diagnosis is set using the EDE [38].

### The mini-international neuropsychiatric interview (MINI)

Present psychosis and depression, suicidal risk and abuse of alcohol, medicine and/or narcotics is assessed by a psychiatrist or attending physician at the patient’s initial assessment interview using the Mini International Neuropsychiatric Interview (MINI) [39]. MINI is a short structured diagnostic interview compatible with international diagnostic criteria, including the International Classification of Diseases (ICD-10) and the Diagnostic and Statistical Manual of Mental Disorders (DSM-IV) [39]. We are using a modified version of the authorized Danish version of the MINI 5.0. We have included the suicidality module from version 6.0.0 and have modified the alcohol dependence and/or alcohol abuse module to Danish norms. Previous studies of the psychometric properties of MINI have shown a good sensitivity, specificity, reliability, and validity [39].

### Standardized assessment of personality (SAPAS)

The possible presence of a DSM-IV recognized personality disorder is assessed with SAPAS [40] at the trial assessment interview. We are using a researcher-rated version of the questionnaire, which consists of eight dichotomously rated questions about the patient’s personality. A previous study indicated that a score of 3 or 4 on the SAPAS correctly identified the presence of a personality disorder in over 80% of 60 psychiatric in- and outpatients [40]. The same study reported that the internal consistency, test-retest reliability, sensitivity, and specificity were acceptable.

### Eating disorder examination (EDE) interview

Eating disorder symptoms are measured with the EDE interview [38,41]. The eating disorder examination is a widely used interview in eating disorders research and is considered ‘the gold standard’ in diagnosing eating disorders. We use the authorized Danish version of the eating disorder examination (EDE) interview (version 12), including the binge eating disorder module from version 16, as the secondary outcome measure and as a diagnostic tool [38,42].We use the global score to compare the groups post treatment.

A high degree of inter-rater reliability, internal consistency and discriminant validity has been reported [43]. The interviews are performed by five interviewers trained by an experienced clinician and researcher. The training consisted of two days at six hours per day.

### The outcome rating scale (ORS)

On the ORS [17], patients assess their wellbeing during the last week. The use of the ORS is described in the section about the feedback intervention.

### The symptom check list (SCL-90R)

Psychological problems and symptoms of psychopathology are measured with the authorized Danish version of the SCL-90R [44,45]. The SCL-90R is a multidimensional patient-reported questionnaire for measuring psychological distress or the degree of affective distress [44-46]. The Global Severity Index (GSI), which is the global score covering all 90 items, will be used in the trial. Internal validity has been tested and found acceptable for all subscales, besides psychoticism [46].

### Sheehan disability scale (SDS)

Functional impairment is measured with the SDS [47]. The SDS is a patient-rated measure of functional disability in work, social life, and family life. The SDS is a composite of three self-rated items of family, work and social impairment in the previous two weeks [48]. Studies of the psychometric properties of the SDS have demonstrated an acceptable reliability, sensitivity to change and construct validity [48].

### WHO-five wellbeing index (WHO-5)

Psychological wellbeing is measured with WHO-5 [49]. The WHO-5 consists of five items that cover positive mood (feeling in good spirits, feeling relaxed), vitality (being active and waking up fresh and rested), and being interested in things. The time frame is the past two weeks and a high score indicates good wellbeing, with a low score indicating the opposite [49]. Studies have found the scale to have a good reliability and excellent validity [50].

### Suicide and self-harm

To assess the occurrence of self-harm behaviour, we have selected seven questions from the Self-Harm Inventory (SHI) [51]. The SHI is in its full length a one-page, 22-item, yes/no questionnaire that explores respondents’ history of self-harm. Each item is preceded by the phrase, ‘Have you ever intentionally, or on purpose…’ followed by (in our modified version): 1: ‘Cut yourself’; 2: ‘Burned yourself’; 3: ‘Hit yourself’; 4: ‘Banged your head’; 5: ‘Scratched yourself’; 6: ‘Prevented wounds from healing’; 7: ‘Attempted suicide’. The time frame is during the treatment period and the questions are presented orally by the assessor at the end of intervention. A recent study comparing six commonly used scales on deliberate self-harm demonstrated that the SHI was psychometrically sound [52].

### Follow-up assessments

A follow-up assessment will be performed within three years after the end of the intervention. The scores of all secondary and explorative outcome measures in the two groups at the longest follow-up (up to 36 months) are compared. See Table 2 for the method and timing of the assessment.

**Table 2:** Method and timing of assessment

	**1. Initial assessment (clinical routine)**	**2. Psychiatric and somatic assessment (clinical routine)**	**3. Trial assessment**	**4. Start of treatment**	**5. During treatment**	**6. End of treatment**	**7. Follow-up**
Sociodemographic data	X						
Eating Disorder Examination (EDE)			X			X	X
Mini International Neuropsychiatric Interview (MINI)		X					
Standardized Assessment of Personality – Abbreviated Scale (SAPAS)			X				
Sheehan Disability Scale (SDS)			X			X	X
Symptom Check List (SCL-90-R)	X					X	X
The Outcome Rating Scale (ORS)				X	X		X
WHO-Five Well Being Index (WHO-5)	X					X	X
Questions about self-harm and suicide						X	X

### Procedures

#### ***Trial conduct***

This trial will be conducted in compliance with the protocol approved by the regulatory authorities: The Regional Ethics Committee of the Capital Region (journal number H-3-2011-151) and the Danish Data Protection Agency (journal number 2007-58-0015), and according to good clinical practice and the Declaration of Helsinki in its latest form. No substantial deviation from the protocol will be implemented without the prior review and approval of the regulatory authorities except where it may be necessary to eliminate an immediate hazard to trial participants.

### Randomization and blinding

The Copenhagen Trial Unit (CTU) will be responsible for the central randomization. They have no clinical involvement in the trial. Randomization is carried out according to a computer-generated allocation sequence with a varying block size kept unknown to the investigators. After each patient is assessed to be eligible for the trial, the trial secretary will call CTU, provide a personal pin code and receive the randomization result.

The randomization is stratified for eating disorder diagnosis (BN, EDNOS or BED) and treatment type (basic or elaborate). Patients and therapists are naturally aware of the patient allocation but secondary outcome assessment (EDE), statistical analyses, and drawing of conclusions will be done with the blind intact. Blinding is maintained by instructing the intervention team to withhold patient information from the research team. The research team has no clinical contact with the patients from assessment to end of intervention, and the assessors have no knowledge about the patient’s randomization status at the end of intervention. In order to prevent the risk of bias, such as when therapists are assessing patient withdrawal, we have a blinded assessor counting the attendance rate and checking each patient’s medical file. In order to prevent actual bias, the research team is not present in supervision or when the therapists are discussing patients at conferences.

### Ethical considerations

This trial is carried out in order to improve treatment for patients with eating disorders. If the results support our hypothesis, the addition of feedback can have the potential of improving treatment outcome in diagnoses other than eating disorders. The control group receives a standardized treatment based on recommendations from the Danish National Board of Health [2] and on guidelines for treating eating disorders in the Capital Region of Denmark [29].

### Risks and benefits

There are no known or obvious risks concerning participation in this trial. The only inconvenience is that participants are required to spend more time on assessment than in clinical routine assessment (filling out questionnaires as well as attending an extra assessment interview pre and post treatment). The assessment provides additional information about the patient’s condition for the intervention team to use in the treatment process. Rating the feedback scales before and after each session can put an extra strain on patients and they are offered assistance in this process. The benefits are estimated to outweigh the risks.

### Statistical plan and data analysis

#### ***Sample size estimation***

We are planning a trial of independent experimental participants and control participants randomized at a 1:1 ratio. Unpublished data (Stolpegaard Psychotherapy Centre, routinely collected data) from 138 patients with eating disorders at Stolpegaard Psychotherapy Centre indicate that the mean number of attended treatment sessions is 13.58 and the SD is 5.82 sessions. We expect to find that the participants in the experimental group attend at least three more sessions than the participants in the control group. Using a SD of six sessions, we need to include 64 participants in each group (total 128) to be able to reject the null hypothesis that number of attended treatment sessions in the experimental and control group is equal with probability (power) 80%. The type I error probability associated with the test of this null hypothesis is 5%. We also estimated the sample size using a power of 90%. This resulted in a total of 170 participants (2 × 85 participants). We therefore plan to recruit a minimum of 128 participants, and in order to reduce the risk of type II error, we will aim to recruit up to 170 participants, if possible in the 14 months recruitment period. Power and sample size calculations have been made using the PS Power and Sample Size Calculations program version 3.0.14 [53,54] (Dupont & Plummer, Nashville, USA).

### Power calculations

We have conducted power calculations for the majority of the secondary and exploratory outcomes (Table 3). Based on unpublished calculations of 176 to 263 previous patients with eating disorders in routine care at Stolpegaard Psychotherapy Centre, the SDs of their post-treatment responses to SCL-90-R and WHO-5 were used. Since the EDE, ORS and SDS are not previously used at Stolpegaard Psychotherapy Centre, power calculation is based on norms from previous studies [55-57].

**Table 3:** Power calculations

**Outcome**	**Minimal relevant difference (points)**	**SD (points)**	**Risk of type I error**	**Power (N = 128)**	**Power (N = 170)**
Eating Disorder Examination (EDE)	0.5	1.33	5%	55.9%	68.3%
Outcome Rating Scale (ORS)	4.5	9.5	5%	75.8%	86.7%
Symptom Check List (SCL-90-R)	0.3	0.67	5%	71.0%	82.8%
WHO-Five Wellbeing Index (WHO-5)	2.5	5.6	5%	70.8%	82.5%
Sheehan Disability Scale (SDS)	3.5	7.1	5%	79.1%	89.2%

### Statistical analysis plan

The analyses are intention–to-treat analyses using a two-sided significance test at 5%. All analyses will be conducted blinded with the two intervention groups coded as A and B.

### Outcomes

The primary outcome is the rate of attendance. It is defined as the number of group sessions the patient has attended over the number of planned sessions (20 or 25 sessions). If at group session # n (n <20) the therapist decides that the patient needs not attend further sessions, the number of planned sessions is set equal to n. When a patient discontinues treatment early, the therapists decide whether this is in accordance with the treatment plan (categorized as ‘planned withdrawal’ or ‘unplanned withdrawal’).

In the analysis of the rate data, the model fit of a Poisson model and a negative binomial model both with offset equal to log (number of planned sessions) are compared and the best fitting model is chosen provided it fits the data reasonably well. In the analysis of continuous outcomes the univariate general linear model is used. If the assumptions of a regression are not fulfilled, a non-parametric test is used (Mann-Whitney).

All outcomes are analyzed using regression analyses, including the binary intervention indicator. The primary results are those adjusted by the protocol-specified stratification variables and (where measured) the baseline value as covariates. In an exploratory analysis the analyses will be repeated with the categorical variable ID-group (identity code of the group) as an additional covariate, and it will be tested if the outcome depends on the treatment group. Then it will be tested if ID-group and the intervention indicator interact. If so, subgroup analyses of the various treatment groups will be conducted.

### Multiplicity

The primary and the secondary outcome are tested in that order each at the 5% level of significance. If the first test is not significant at the 5% level the null hypothesis of the secondary outcome is accepted without test.

### Missing values

Missing values of the secondary outcome and/or covariates included in its analysis are imputed using multiple imputation (SPSS), if the percent of missing patients is >5% and *P* of Little’s test is <5%. The result of the analysis of the imputed datasets is then the primary result.

The assumption of multiple imputation is that the data are missing at random, i.e., that for a given quantity with missing values, the distribution of observed values of this quantity conditional on values observed of other quantities is the same as the conditional distribution of the values planned to be observed of the quantity, but missing. If this condition is not fulfilled, the results of the multiple imputation will be biased. To obtain the range of plausible bias that may result if the MAR (missing at random) condition is not fulfilled, we will do the following two analyses using single imputations of the outcome performed as a sensitivity analysis: 1) missing values in group A are imputed by the minimum value observed in the material, while missing values in group B are imputed by the maximum value observed, and the two groups are compared; and 2) vice versa.

## Discussion

Attendance to psychotherapy is a prerequisite for the therapy to have an effect. However, a large proportion of patients end treatment prematurely, which has been associated with poorer treatment outcomes [5,6]. In this trial, we hypothesize that integrating patient feedback in the psychotherapy process will increase attendance and patient outcome. This is the first randomized trial of the effect of feedback on group psychotherapy for patients with eating disorders. Below, we discuss some of the potential limitations and strengths of the F-EAT trial.

The trial is subject to at least four potential limitations. First, although the trial was designed in order to minimize the risk of bias [58-60] and the risk of random errors [58], most outcomes are at risk of being assessed with some bias, as only the secondary outcome measure (EDE) is possible to blind [58-60]. Previous research has indicated that non-blinded assessors tended to be more optimistic about patient outcome when compared to blinded assessors [61]. Blinding is thus used whenever possible and data will be analyzed according to the intention-to-treat principle. We employed central randomization with an allocation sequence stratified for prognostic factors [58-60].

Second, the therapists are trained and supervised by an engaged trainer and became excited about the feedback method. The trainer is the only certified trainer in Denmark and is herself involved in the ICCE. It is possible that, as she believes in the benefits of feedback, this might influence the therapist allegiance. The potential allegiance bias was part of our considerations beforehand, however it was important to us that the therapists received proper training and continuing supervision in the feedback method. The alternative would be to collaborate with a less experienced trainer and/or have less training. This solution could imply that the therapists failed to comply with the method or refused to use it, and would pose a greater risk to the trial overall since it would compromise data collection.

Third, the fact that most therapists provide treatment both in an experimental and a control group can cause a contamination between the groups. However, neither the participants nor the therapists in the control groups are informed of the patients’ progress, and we therefore hypothesize that any contamination will be minor.

Fourth, the participants in the control groups use a paper version of the ORS while participants in the experimental groups use a tablet computer to mark their score. These two different versions of the ORS might differ in psychometric properties and thus influence the results [62]. This difference is nevertheless an important premise of the trial design because we hypothesize that the available feedback (from the management system on the tablet computer) will improve attendance and outcome in the experimental groups when compared to the control groups.

We would also like to emphasize at least three possible strengths of the trial. First, we planned for the participants to be as similar as possible to the patients treated in routine care. We have therefore added as few inclusion and exclusion criteria as possible in addition to the ones normally applied when patients are referred to treatment at Stolpegaard Psychotherapy Centre. Accordingly, the findings of the trial should have a wide generalizability.

Second, by using multiple outcome measures (patient-, therapist-, researcher-rated measures, and objective measures), we hope to discuss outcome in broader terms. Previous research comparing patient-reported outcomes and therapist-reported outcomes has indicated that therapists are more positive than patients in their evaluation of symptom relief [63].

Third, we expect to widen the scope of this relatively new area of research by including more clinically distressed patients in routine care. Compared to participants in the reviewed trials, participants in our trial are clinically less diverse, especially concerning symptom severity, sex, age, cultural and economic background, as well as intervention. The homogeneity might reduce the risk of bias and thus enable us to draw more certain conclusions.

## Trial status

The trial is currently in the recruitment phase. The first participant was included and randomized on 28 August 2012.

## Abbreviations

AN: Anorexia Nervosa; BED: Binge Eating Disorder; BMI: Body Mass Index; BN: Bulimia Nervosa; CTU: Copenhagen Trial Unit; DSM: Diagnostic and Statistical Manual of mental disorders; EDE: Eating Disorder Examination interview; EDNOS: Eating Disorder Not Otherwise Specified; ETR: Expected Treatment Response; FIT: Feedback Informed Treatment; GSI: Global Severity Index; GSRS: Group Session Rating Scale; ICD: International Classification of Diseases; ICCE: International Center for Clincial Excellence; MINI: Mini International Neuropsychiatric Interview; OQ System: Outcome Questionnaire System; ORS: Outcome Rating Scale; PCOMS ICCE: Partners for Change Outcome Management System: International Center for Clinical Excellence; SAPAS: Standardized Assessment of Personality – Abbreviated Scale; SCL: Symptom Check List; SD: Standard Deviation; SDS: Sheehan Disability Scale; SHI: Self-Harm Inventory; SRS: Session Rating Scale; WHO: World Health Organization.

## Competing interests

The authors declare that they have no competing interests.

## Authors’ contributions

The trial design was developed by SP, ML, MW, JL and AD. AD drafted the manuscript, which was carefully revised, edited, and discussed by SP, ML, MW and JL. All authors read and approved the final manuscript.
